# Genetically predicted obstructive sleep apnea is causally associated with an increased risk for periodontitis

**DOI:** 10.1186/s12903-023-03338-8

**Published:** 2023-10-06

**Authors:** Zhongqian Mi, Nan Meng, Yitao Zhang, Qianqian Wang, Shan Song, Rui Cheng, Xiaojiang Xu, Jinhua Gao, Feiyan Yu, Xiuyun Ren

**Affiliations:** 1 Shanxi Medical University School and Hospital of Stomatology, 030001 Taiyuan, China; 2 Shanxi Province Key Laboratory of Oral Diseases Prevention and New Materials, Taiyuan, China; 3 Department of Rheumatology, The Second Hospital of Shanxi Medical University, Taiyuan, China

**Keywords:** Periodontitis, Obstructive sleep apnea, Causality, Mendelian randomization

## Abstract

**Background:**

Although obstructive sleep apnea (OSA) and periodontitis are associated, whether this association is causative is uncertain.

**Methods:**

We conducted a bidirectional Mendelian randomization (MR) analysis using data from publically accessible genome-wide association studies. The single-nucleotide polymorphisms (SNPs) for OSA were derived from 16,761 cases and 201,194 controls. The pooled data of periodontitis association involved up to 17,353 individuals. Disease-associated single-nucleotide polymorphisms were selected as an instrumental variable at the genome-wide significance level (*p* < 5.0 × 10^− 6^). Subsequently, the causal effects were estimated using three different methods: inverse variance weighting (IVW), MR-Egger, and weighted median. Then, these causal estimates were expressed as dominance ratios [odds ratio (OR)].

**Results:**

The MR analysis revealed that genetically determined OSA promotes the development of periodontitis [ IVW OR = 1.117, 95% confidence interval (CI) = 1.001–1.246, *p* = 0.048). Furthermore, no causal effect of genetically predicted periodontitis on OSA was noted in the reverse MR analysis (IVW OR = 1, 95% CI: 0.95–1.06, *p* = 0.87). The trend in results from the MR-Egger regression and weighted median (WM) was consistent with that in results from the IVW method. The robustness of the results was confirmed by the sensitivity analysis.

**Conclusions:**

In summary, the results of our MR investigation suggest an association between OSA and periodontitis, proposing that early screening and treatment of OSA is beneficial for the prevention and prognosis of periodontitis.

**Supplementary Information:**

The online version contains supplementary material available at 10.1186/s12903-023-03338-8.

## Introduction

Periodontitis is a chronic inflammatory disease associated with dysbiotic polymicrobial communities and inflammatory, environmental, and genetic susceptibility factors [1]. The major clinical manifestations of periodontitis are the growth of deep periodontal pockets and the degradation of alveolar bone, eventually causing tooth loosening and loss [2]. The degree of susceptibility to periodontitis varies significantly across individuals [3]. Moreover, systemic disorders, including diabetes mellitus [4, 5], osteoporosis [6], and respiratory diseases [7], can weaken or impair the resistance of periodontal tissues to external stimuli and thus support the development and progression of periodontitis. Obstructive sleep apnea (OSA) is the most prevalent type of sleep breathing disorder; it is marked by the recurrent, periodic partial, or total collapse of the upper airway during sleep [8, 9]. The main clinical symptoms of OSA are intermittent hypoxia, hypoxemia, and sleep fragmentation [10]. Moreover, intermittent hypoxia in patients with OSA can enhance the release of inflammatory mediators such as tumor necrosis factor-κB, interleukin (IL)-6, and IL-1β, which can lead to a systemic inflammatory response [11]. OSA-induced inflammatory responses along with genetic and other factors can worsen existing inflammatory diseases.

The association between OSA and periodontitis is well-known [12, 13]. Moreover, several case-control studies have shown a higher prevalence of periodontitis in patients with OSA [14–16]. A meta-analysis including 30,994 subjects showed a significantly higher prevalence of periodontitis in patients with OSA than in those without OSA [odds ratio (OR) = 2.17; 95% confidence interval (CI): 1.66–2.83) [17]. Another meta-analysis including nine international studies showed a similar conclusion that patients with OSA are 1.56 times more likely to develop periodontitis than those without OSA (OR = 1.56; 95% CI: 1.06–2.06) [18]. Furthermore, OSA is associated with more severe periodontal disease and higher levels of IL-6 in the saliva and appeared to change the bacteria tested in plaque [19]. Given the possibility of an association between OSA and periodontal disease, few studies showed that there was no significant association between them [20, 21]. Differences in study design, periodontal evaluation methods, or demographic and societal factors can be the reason for the discrepancy in the reported results; thus, the causal relationship between OSA and periodontitis is still controversial.

The Mendelian randomization (MR) analysis is a method that uses genetic variation as an instrumental variable (IV) for assessing the causality relationship between illness exposure and outcome. MR is based on Mendel’s Law of Heredity (alleles are randomly assigned to offspring gametes at the time of gamete formation) [22] and can avoid potential unmeasured confounding factors and reverse causality [23], making it more convincing and reliable than traditional observational studies. Randomized controlled trials for studying the association between OSA and periodontitis results are virtually impossible, making it difficult to conclude the cause and effect. MR studies are increasingly being used as an alternative to randomized controlled trials for enhancing causal conclusions regarding relationships in observational research.

In this study, we aimed to evaluate the causal effect between OSA and periodontitis using MR analysis. We used the genetic summary data from two separate, sizable genome-wide association studies (GWASs) in the present study. Herein, single-nucleotide polymorphisms (SNPs) associated with risk factors were used as IVs in a two-way MR analysis. This study provides solid proof when developing intervention strategies and enables the development of innovative approaches for the treatment and prevention of periodontitis.

## Materials and methods

### Study design and data sources

**Fig. 1:**
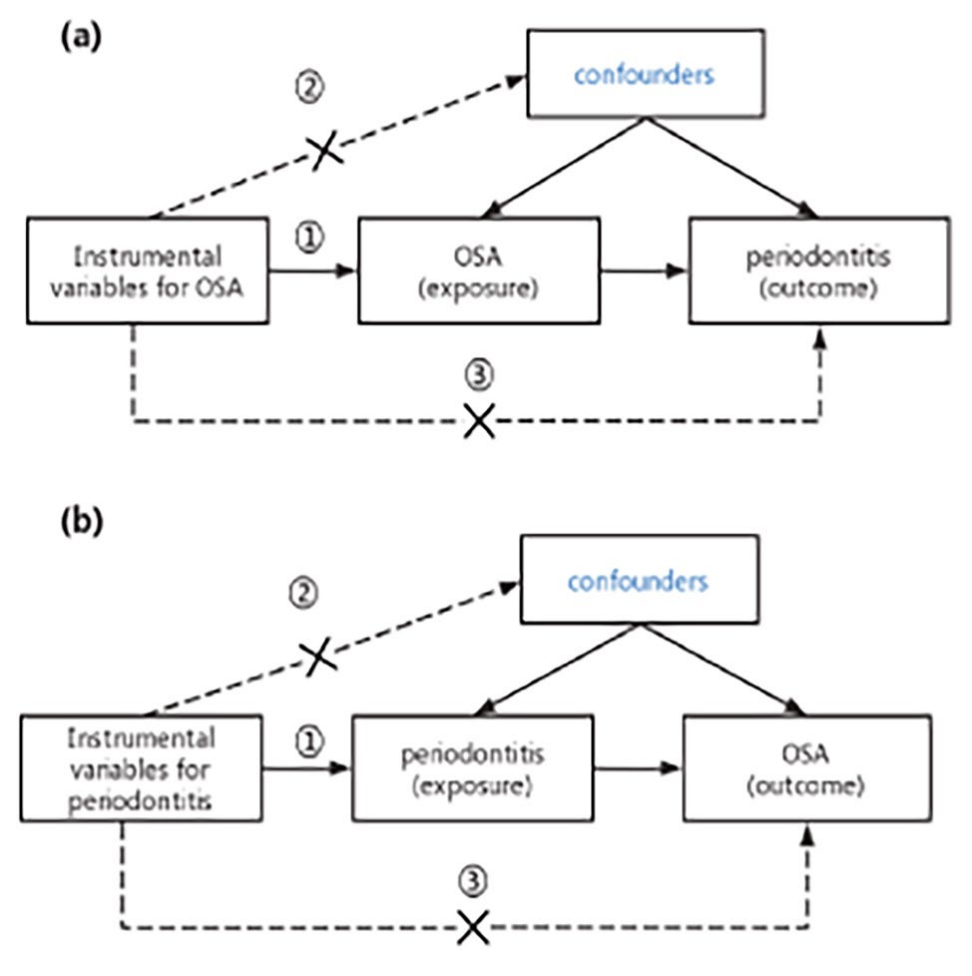
Diagram of Mendelian randomization (MR) study design. (**a**) The evaluation of the causal effect of OSA on periodontitis. (**b**) The evaluation of the causal effect of periodontitis on OSA. (1) Each genetic variation (SNP) is linked to exposure (disease or phenotypic features); (2) SNPs are not associated with unmeasured confounders; and (3) SNPs can only affect the outcome via exposure; they cannot affect the outcome through other routes. OSA, obstructive sleep apnea; SNP, single nucleotide polymorphism

To obtain reliable results, IVs must satisfy three key assumptions in the MR analysis process: [1] Genetic variation should be significantly associated with exposure; [2] IVs should not be associated with any confounding factors affecting the exposure and outcome; and [3] IVs should affect outcome only indirectly through exposure factors and not through other pathways (no horizontal pleiotropy). Figure 1 shows an overview of the study’s design.

The GWAS summary data are publicly available for our study. The IEU GWAS website was searched using the phrase “Sleep Apnea” and chose the GWAS summary data with ID “finn-b-G6 SLEEPAPNO” as the genetic variant linked to OSA. Data from this GWAS analysis, including 16,761 OSA cases, and 201,194 controls, were taken from the FinnGen consortium (R6). The diagnosis of OSA was made by the International Classification of Diseases (ICD-10: G47.3, ICD-9: 3472 A), which is based on patient reports of symptoms, clinical examination, and sleep registry using the apnea–hypopnea index or respiratory event index of 5/hour. Age, sex, genotyping chip, genetic relationship, and the first 10 main components of the analyses were all taken into account. In total, 16,383,314 SNPs were available for investigation. The periodontitis summary statistics were obtained from the most recent meta-analysis of the GWAS of the Gene-Lifestyle Interaction in the Dental Endpoints Consortium. To date, this is the largest study, with 17,353 clinically diagnosed cases and 28,210 controls. Cases of periodontitis are defined by either the Centers for Disease Control and Prevention/American Academy of Periodontology definitions(CDC/AAP) [24] or the Community Periodontal Index (CPI). GWAS analyses were conducted on participants with European ancestry. In all of these summary genetic association estimates, the source articles contain more information on the population characteristics and particular trait definitions. All the GWAS data involved in this study complied with the Declaration of Helsinki published in 1975 (amended in 2013), and each study received ethical approval from the local institutional ethics committees and informed consent from all patients.

### Instruments selected for genetic analysis

SNPs were classified as IVs in the current investigation. For the first hypothesis, candidate IVs were created using summary statistics of exposure-associated SNPs with genome-wide significance (*p* < 5e^− 6^). For each exposure, we used the clumping technique with R^2^ < 0.001 and a window size of 10,000 kb to filter SNPs that were in significant linkage disequilibrium to guarantee independence among instrumental factors. Then, SNPs with minor allele frequencies of < 0.01 were removed. The F statistic was used as a measure of the strength of the IV of SNPs, calculated separately and cumulatively for each SNP via the formula F = R^2^ × (N − 2)/(1 − R^2^). The F-statistics for each of these IVs needed to be > 10, indicating that they fulfill the strong correlation requirement of MR. Considering the second hypothesis of MR, we used the PhenoScanner database(http://www.Phenoscanner.cam.ac.uk/)to identify SNPs with possible associations with confounders and remove them. After several rigorous screenings, the remaining SNPs were considered eligible IVs.

### MR analyses

To investigate the potential causal inferences between OSA and periodontitis, inverse variance weighting (IVW) [25], MR-Egger regression [26], and weighted median (WM) [26] were the three MR techniques used. The IVW method is the most commonly used method for MR analysis and is comparable to a weighted linear regression of the correlation between IV and outcome. The IVW method uses meta-analysis to combine the Wald estimates of each SNP for obtaining two-sample MR estimates of the association between OSA and periodontitis to evaluate causality [27]. For estimating causality, the MR-Egger regression and WM method are also comparable to the IVW method. The slope of the MR-Egger represents the potential causal effect, on top of its intercept term, which facilitates the assessment of horizontal multiplicity [28]. When there is evidence of pleiotropy, MR-Egger regressions are preferred. WM allows consistent analysis of multiple genetic instruments by calculating a single WM estimate; the results can provide reliable causal estimates even if up to 50% of SNPs are invalid genetic instruments [26]. MR estimates are reported as the odds ratio (OR) of results increased per unit ln (OR) of exposure. It should be noted that MR estimation associated with binary exposure (unlike continuous exposure) is more effective for identifying the presence of causal effects than quantifying the magnitude of causal effects [29].

### Pleiotropy and sensitivity analysis

For the third hypothesis, each IV could only affect the outcome through exposure factors and not through other pathways. MR-Egger regression was performed to assess the likelihood of horizontal pleiotropy. The mean pleiotropic effect of IV was characterized by the intercept term of the MR-Egger regression. To correct horizontal pleiotropy by removing potential outliers, the MR-PRESSO was used as part of the pleiotropy analysis [30]. The Cochran’s Q test was used to verify the value of heterogeneity between the causal estimates of each SNP for the IVW and MR-Egger methods. There was no heterogeneity between IVs if the Q statistic was *p* > 0.1. Furthermore, a “leave-one-out” sensitivity analysis was performed to determine whether the potential effect of an SNP on the causal estimates was caused by a particular SNP and to verify the robustness and consistency of the findings.

“Two SampleMR” [31] packages were used for all studies. A causal relationship was presumed to exist if an observed *P* < 0.05 supported it statistically.

## Results

### Selection of IVs for OSA and periodontitis

SNPs strongly associated with OSA in our primary analysis were selected at the genome-wide significance level (*p* < 5e^− 6^). To ensure that exposed instrumental SNPs were not in a state of linkage disequilibrium, studies were performed by clustering European samples from 1000 genomic projects (R^2^ < 0.001, window size = 10,000 kb). Avoiding strong linkage disequilibrium instrumental SNPs may cause biased results. We obtained 31 SNPs in the table of exposure factors and outcome variables. The F-statistics for these IVs (27.25125–85.66920) were all > 10, demonstrating that there was no weak instrumental bias affecting the estimation of causal effects. SNPs with minor allele frequencies of < 0.01 were also eliminated. Considering that body mass index, obesity, smoking, alcohol consumption, and type 2 diabetes are common confounders of periodontitis, we excluded five SNPs associated with the abovementioned confounders (rs10938398, rs4837016, rs72632980, rs9937053, and rs6021831). For the MR analysis of OSA causally connected with periodontitis, 24 SNPs potentially associated with OSA were chosen as genetic tools (Supp. Table S1).

### Causal impact of OSA on periodontitis

**Fig. 2:**
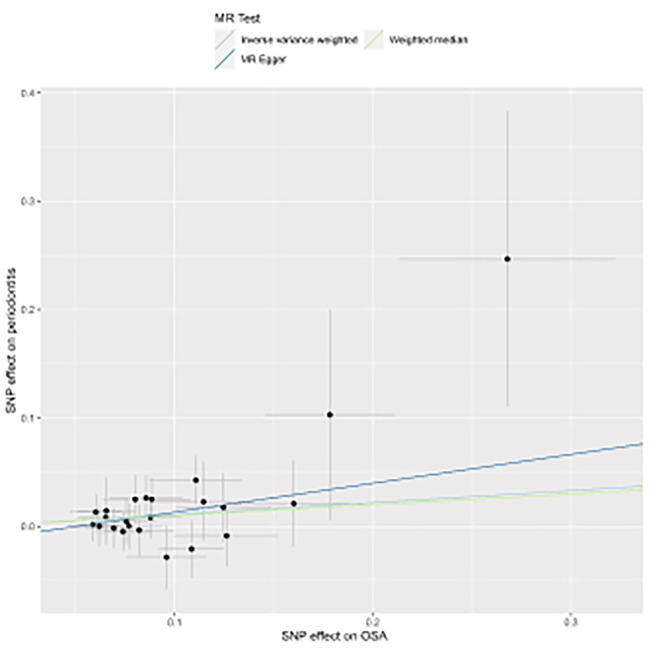
Scatter plot depicts the causal estimates of the effect of OSA on periodontitis. Each point in the scatter plot represents an SNP. The effect of the same SNP on exposure is placed on the horizontal axis, and the effect on outcome is placed on the vertical axis. The vertical and horizontal lines show the 95% confidence interval (CI) for each SNP. At this point, the slope of the solid line in the plot is each Mendelian randomization (MR) estimate. OSA, obstructive sleep apnea; SNP, single nucleotide polymorphism

Various MR approaches were used to confirm the possible link between OSA and periodontitis. The IVW method was used to assess a positive association between genetically predicted OSA and periodontitis, revealing that the risk of periodontitis was 1.117 times higher in individuals with OSA than in those without OSA (OR _IVW_ = 1.117, 95% CI = 0.001–1.246, *p* = 0.048). Furthermore, MR assessments using MR-Egger estimates (OR _MR−Egger_ = 1.305, 95% CIs = 0.859–1.983, *p* = 0.225) and WMs (OR _WM_ = 1.105, 95% CIs = 0.95–1.281, *p* = 0.186) revealed generally consistent directions of effect, although they were typically rarely statistically significant, owing to the lower power of these two approaches (Table 1; Fig. 2).

**Table 1:** Summary statistics for MR analysis of the potential causal effect of periodontitis and OSA

Exposure	Outcome	Methods	N. SNP	Beta	SE	*P*.value	OR	95%CI	
OSA	Periodontitis	IVW	24	0.11	0.06	0.04	1.12	1.00	1.25
		MR- Egger	24	0.27	0.21	0.23	1.31	0.86	1.98
		WM	24	0.08	0.08	0.19	1.11	0.95	1.28
Periodontitis	OSA	IVW	7	0.005	0.028	0.87	1.00	0.95	0.95
		MR- Egger	7	0.001	0.033	0.98	1.00	0.94	0.94
		WM	7	0.009	0.034	0.78	0.99	0.93	0.93

OSA, Obstructive sleep apnea; IVW, inverse variance weighted; WM, Weighted Median; N.SNPs, number of SNPs used in MR; SE, standard errorOR, odds ratio; CI, confidence interval; *P.*value < 0.05 was considered as an indication of statistical significance

### Sensitivity analysis to support the hypothesized causal relationship between OSA and periodontitis

**Fig. 3:**
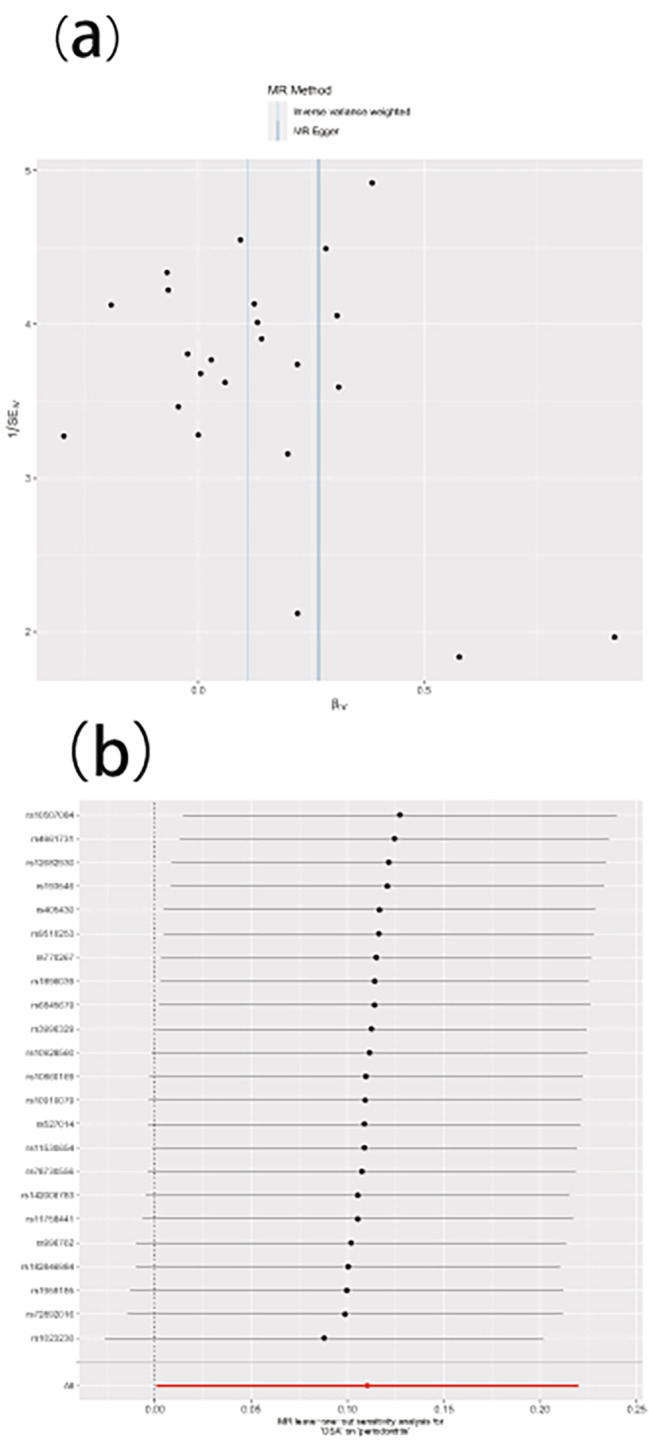
The funnel plot (**a**) and leave-one-out plot (**b**) in the sensitivity analysis of the periodontitis-OSA relationship are presented. The funnel plot assesses the possible heterogeneity in the estimates. Each point indicates the inverse standard error corresponding to the individual causal estimates. The mean causal effect of all combinations of IVs (βIV) is indicated on the X-axis by inverse variance weighting (solid line) and the Mendelian randomization (MR)-Egger method (dashed line). The Y-axis indicates the inverse standard error of the estimated causal effect for each single nucleotide polymorphism (IVs). B The leave-one-out plot present how the causal estimates (point with horizontal circle) for the effect of OSA on periodontitis were influenced by the removal of a single variant. The bars indicate the confidence interval of MR estimates. OSA, obstructive sleep apnea; SNP, single nucleotide polymorphism

The Cochran’s Q statistic found no proof of heterogeneity in causal inferences (MR-Egger *p* = 0.938 and IVW *p* = 0.943) (Table 2; Fig. 3); the intercept term for the MR-Egger regression was calculated, which showed no horizontal pleiotropy among the seven selected SNPs (intercept = − 0.013; standard error 0.018, *p* = 0.456). The results of the leave-one-out analysis recommend that the association between OSA and periodontitis risk is not driven by individual SNPs, demonstrating that the MR results are robust and reliable (Fig. 3).

**Table 2:** The heterogeneity of individual SNPs

		Heterogeneity					
		MR-Egger			IVW		
Exposure	Outcome	*Q*	*df*	*P*-value	*Q*	*df*	*P*-value
OSA	Periodontitis	12.06	21	0.94	12.64	22	0.94
Periodontitis	OSA	3.62	5	0.61	3.66	6	0.72

OSA, Obstructive sleep apnea; IVW, inverse variance weighted; WM, Weighted Median *P*. value < 0.05 was considered as an indication of statistical significance

### Causal effects of periodontitis on OSA

Using the same method as forward MR analyses, SNPs for reverse MR were obtained. After taking into consideration linkage disequilibrium (R^2^ < 0.001, window size = 10,000 kb), we used Seven SNPs that were individually and substantially associated with periodontitis. All F-statistics for the IVs used to measure periodontitis-related traits were > 10 (124.0636–2638.8544); there was no obvious bias caused by weak instrumental factors. Moreover, the elimination of several SNPs with minor allele frequencies. Considering the potential impact of confounding, one SNP associated with BIM was further excluded. The remaining seven SNPs served as a tool for our MR analysis (Supp. Table S2). According to the main findings of IVW, no statistical evidence linking an increased incidence of OSA to an increased risk of periodontitis was noted (OR = 1.00, 95% CI: 0.95–1.06, *p* = 0.87). Furthermore, consistent findings were obtained using the MR-Egger (OR = 1.00, 95% CI: 0.94–1.07, *p* = 0.98) and WM (OR = 0.99, 95% CI: 0.93–1.06, *p* = 0.78). A Cochran’s Q statistic evaluation of the SNPs revealed no evidence of heterogeneity between them (MR-Egger *p* = 0.605 IVW *p* = 0.723) (Table 2). The results of the MR-Egger intercept test ruled out any directional pleiotropy (intercept = 0.002; standard error = 0.009, *p* = 0.850). The leave-one-out sensitivity analysis showed that no particular SNP was significantly responsible for the association between OSA and periodontitis (Supp. Fig.S1-S3).

## Discussion

To our knowledge, this study is the first that uses a sizable GWAS dataset with MR analysis for assessing the causal link between OSA and periodontitis. Our findings provide credibility to the idea that OSA causatively affects periodontitis. A reverse MR study was also conducted, but no evidence suggested periodontitis as a cause of OSA.

Observational research has recently shown that OSA may play a critical role in the development and progression of periodontitis. A case-control study using conditional logistic regression analysis on 7673 patients with OSA and 21,963 controls demonstrated a relationship between OSA and previously identified periodontitis [32]. Similar conclusions have been obtained by stratification studies conducted for different age groups. Based on the findings of a cross-sectional study conducted in Korea, patients with OSA aged ≥ 55 years have more than double the risk of developing chronic periodontitis than healthy controls [13]. Another large population-based study including Hispanic/Latino adults further showed that breathing disorders during sleep and severe periodontitis in young adults are positively associated [13]. Furthermore, results from observational studies in several countries, including Jordan, India, and the United States, have shown that OSA and a higher risk of periodontitis are associated, suggesting that OSA is a potential predictor of periodontitis [33–35]. Similarly, a meta-analysis further confirmed this [17, 18, 36, 37]. Our MR research is in line with prior findings and supports the hypothesis that the increased incidence of periodontitis owing to OSA is driven by a causative impact.

To clarify the association between OSA and periodontitis, several explanations have been proposed in prior research. First, mouth breathing is a potential cause of periodontal diseases. Oral breathing is a habitual behavior of patients with OSA. Prolonged mouth opening may alter the oral environment and hinder the mouth’s natural cleansing processes, facilitating greater colonization of the periodontal microbiota and subsequent development of periodontitis [38, 39]. Second, OSA is often associated with intermittent hypoxia (IH).OSA can encourage transcription factors such as nuclear factor due to intermittent hypoxia, causing increased production of pro-inflammatory cytokines that can aggravate the inflammatory condition of preexisting disease and even start inflammatory illness in the host, thus causing an augmented risk of periodontitis and its severity [40]. In vivo, studies have shown that intermittent hypoxia in OSA might affect immune transcription factors such as host HIF-1, which can considerably increase bone mineral density and modify bone microstructure, which is a possible risk factor for poor autostasis in developing alveolar bone [41, 42]. Furthermore, oxidative stress is an additional factor that contributes to OSA-related periodontal tissue damage. The mechanism of this includes encouraging the generation of reactive oxygen species and oxygen free radicals, leading to the formation of local and systemic inflammatory responses [43]. Sub-gingival plaque and saliva-serum cytokine levels were measured in patients with OSA in a study; OSA was found to be associated with worsening periodontal disease and greater amounts of IL-6 and apelin in the saliva, as well as changed the bacteria that were examined in plaque [11, 15, 19]. Furthermore, by changing the microbial community around the periodontium, OSA can initiate the development of periodontitis. A study using 16 S rRNA sequencing revealed that the species richness and trans-habitat diversity of the salivary microbial community were altered in the OSA group, and coupled with *Prevotella* (a particular periodontal pathogen), it showed an increase in the tendency of OSA in patients [40]. These findings offer fresh insights into the pathophysiology of periodontitis.

We were able to evaluate the effect of OSA on periodontitis more systematically using the two-sample MR design and a sizable data set (16,761 OSA cases and 201,194 controls; 17,353 patients with periodontitis and 28,210 controls). Since SNPs are randomly assigned at the time of inheritance and are independent of confounding factors, bias due to reverse causation or confounding is substantially reduced in the MR designs. The MR procedures can make the case for causal inference stronger than that of observational investigations [25]. Another strength is that the majority of participants recruited in these two separate associations were of European origin, which minimized the effect of population stratification.

Nevertheless, the present study has several limitations. First, in MR, the most widely adopted approach relies on the inference of SNPS identified by genome-wide association studies (GWAS). Given the variations in quality control when conducting individual GWAS, it was difficult to offset the potential confounding bias that would have affected the results. Second, our findings only reflect the life-long impact of OSA on periodontitis. However, the short-term effect of OSA on the risk of periodontitis remains unknown. Third, given our population restriction to European ancestry, the conclusions cannot be generalized to other populations. Additionally, since the research used pooled data, further stratified data analyses based on individual-level data are needed to further examine the effect of the causal link between OSA and periodontitis. Finally, due to the inconsistent definition of periodontitis used in various studies and the numerous challenges faced during disease analysis, GWAS for periodontitis tends to fail to discover consistent SNPs. Future high-quality GWAS are still required to further study the potential etiological role of OSA in periodontitis.

## Conclusion

In conclusion, our study revealed enough evidence to establish OSA as genetic proof of a causative effect of exposure on the higher risk of periodontitis. Moreover, we suggest that early detection and management of OSA can be a new strategy to improve periodontitis in the future. However, the contrary conclusion only yielded little support. Further research is required on the precise mechanisms that contribute to the causal link between OSA and periodontitis.

### Electronic supplementary material

Below is the link to the electronic supplementary material.

Supplementary Material 1

Supplementary Material 2

Supplementary Material 3

Supplementary Material 4

Supplementary Material 5

Supplementary Material 6

Supplementary Material 7

## Data Availability

All data generated or analyzed during this study are included in this published article and its additional material.
