# Supplementary material for: Genetically predicted obstructive sleep apnea is causally associated with an increased risk for periodontitis
Source: BMC Oral Health. 2023 Oct 6;23:723. doi: 10.1186/s12903-023-03338-8 (PMC10559524; doi:10.1186/s12903-023-03338-8)
Supplement: Supplementary file 1 — Supplementary Material 1 [file 12903_2023_3338_MOESM1_ESM.docx]

| Supplementary Table 1 Twenty-four independent SNPs related to OSA | | | | | | | | | | | |
| --- | --- | --- | --- | --- | --- | --- | --- | --- | --- | --- | --- |
| **NO** | **SNP** | **Chr** | **EA** | **OA** | **EAF** | **Exposure:OSA** | | | **Outcome:periodontits** | | |
|  |  |  |  |  |  | **Beta** | **SE** | ***P*** | **Beta** | **SE** | ***P*** |
| 1 | rs1023230 | 13 | T | C | 0.9223 | -0.1107 | 0.0232 | 1.83E-06 | -0.0426 | 0.0225 | 0.05892 |
| 2 | rs10475978 | 5 | C | G | 0.4904 | -0.0577 | 0.0125 | 3.95E-06 | 0.0065 | 0.0154 | 0.6732 |
| 3 | rs10507084 | 12 | T | C | 0.1793 | 0.1085 | 0.0163 | 2.80E-11 | -0.0209 | 0.0263 | 0.4265 |
| 4 | rs10860169 | 12 | G | A | 0.2906 | -0.0653 | 0.0137 | 2.01E-06 | -0.0081 | 0.0158 | 0.6074 |
| 5 | rs10910079 | 1 | T | C | 0.03324 | 0.1601 | 0.0348 | 4.13E-06 | 0.021 | 0.0399 | 0.5986 |
| 6 | rs10928560 | 2 | T | C | 0.1949 | -0.0878 | 0.0158 | 2.80E-08 | -0.0082 | 0.0193 | 0.6699 |
| 7 | rs11530654 | 11 | C | A | 0.2633 | 0.0655 | 0.0141 | 3.54E-06 | 0.0144 | 0.0309 | 0.6401 |
| 8 | rs11758441 | 6 | T | C | 0.378 | 0.0602 | 0.0129 | 2.98E-06 | 0.0132 | 0.0161 | 0.412 |
| 9 | rs12682930 | 9 | T | G | 0.0609 | -0.1262 | 0.0262 | 1.49E-06 | 0.0087 | 0.0291 | 0.7641 |
| 10 | rs142006783 | 16 | C | T | 0.03778 | 0.1783 | 0.0327 | 4.81E-08 | 0.103 | 0.0971 | 0.2892 |
| 11 | rs182846984 | 4 | T | G | 0.01414 | 0.2679 | 0.0544 | 8.36E-07 | 0.2468 | 0.1363 | 0.07021 |
| 12 | rs1896039 | 12 | A | G | 0.5349 | 0.062 | 0.0126 | 9.24E-07 | 0 | 0.0189 | 0.9981 |
| 13 | rs193546 | 14 | A | G | 0.7438 | 0.0739 | 0.0143 | 2.27E-07 | -0.0049 | 0.0175 | 0.7771 |
| 14 | rs1959185 | 14 | A | G | 0.1344 | 0.0856 | 0.0182 | 2.58E-06 | 0.0263 | 0.0211 | 0.2131 |
| 15 | rs3996329 | 7 | A | G | 0.2366 | -0.0757 | 0.0148 | 2.91E-07 | -0.0045 | 0.0209 | 0.8302 |
| 16 | rs405430 | 9 | T | G | 0.7625 | -0.0693 | 0.0146 | 2.10E-06 | 0.0016 | 0.0182 | 0.9301 |
| 17 | rs4961731 | 9 | T | C | 0.8894 | 0.0959 | 0.02 | 1.62E-06 | -0.0285 | 0.0293 | 0.3314 |
| 18 | rs527014 | 1 | T | C | 0.07433 | 0.1246 | 0.0238 | 1.54E-07 | 0.0175 | 0.0319 | 0.5833 |
| 19 | rs6845679 | 4 | T | C | 0.5898 | 0.0588 | 0.0127 | 3.53E-06 | 0.0017 | 0.0156 | 0.9125 |
| 20 | rs72892016 | 18 | A | G | 0.1204 | -0.0885 | 0.0192 | 3.94E-06 | -0.025 | 0.0197 | 0.2042 |
| 21 | rs770267 | 13 | G | A | 0.831 | 0.0769 | 0.0166 | 3.83E-06 | 4.00E-04 | 0.0209 | 0.9829 |
| 22 | rs78730556 | 4 | T | C | 0.06738 | -0.1146 | 0.0251 | 4.82E-06 | -0.0227 | 0.0363 | 0.5315 |
| 23 | rs9510253 | 13 | T | A | 0.1468 | 0.0821 | 0.0176 | 3.08E-06 | -0.0036 | 0.0237 | 0.8801 |
| 24 | rs996762 | 2 | G | C | 0.8239 | -0.0801 | 0.0163 | 8.69E-07 | -0.0249 | 0.0223 | 0.2643 |
| Note:SNP:single nucleotide polymorphism. OSA:Obstructive sleep apnea;EA:effect allele. OA:other allele. | | | | | | | | | | | |
