# Supplementary material for: Genetically predicted obstructive sleep apnea is causally associated with an increased risk for periodontitis
Source: BMC Oral Health. 2023 Oct 6;23:723. doi: 10.1186/s12903-023-03338-8 (PMC10559524; doi:10.1186/s12903-023-03338-8)
Supplement: Supplementary file 2 — Supplementary Material 2 [file 12903_2023_3338_MOESM2_ESM.docx]

| Supplementary Table 2 seven independent SNPs related to periodontitis | | | | | | | | | | | |
| --- | --- | --- | --- | --- | --- | --- | --- | --- | --- | --- | --- |
| **NO** | **SNP** | **Chr** | **EA** | **OA** | **EAF** | **Exposure:OSA** | | | **Outcome:periodontits** | | |
|  |  |  |  |  |  | **Beta** | **SE** | ***P*** | **Beta** | **SE** | ***P*** |
| 1 | rs10143801 | 14 | G | A | 0.3258 | 0.084 | 0.0171 | 8.66E-07 | 0.0103 | 0.0131 | 0.4326 |
| 2 | rs138868497 | 11 | C | T | 0.0103 | -1.6387 | 0.3324 | 8.20E-07 | 0.0173 | 0.0544 | 0.751 |
| 3 | rs151226594 | 11 | G | T | 0.0184 | 0.3671 | 0.0768 | 1.75E-06 | -0.0185 | 0.0434 | 0.6698 |
| 4 | rs4811024 | 20 | G | C | 0.8945 | 0.1337 | 0.0292 | 4.62E-06 | 0.0025 | 0.0247 | 0.919 |
| 5 | rs73155039 | 7 | G | A | 0.0106 | -0.8316 | 0.1757 | 2.22E-06 | -0.133 | 0.0832 | 0.1101 |
| 6 | rs76734229 | 18 | A | G | 0.0737 | -0.1761 | 0.037 | 1.94E-06 | 2.00E-04 | 0.0219 | 0.9929 |
| 7 | rs9954920 | 18 | T | C | 0.3572 | 0.0769 | 0.0163 | 2.37E-06 | -0.0062 | 0.0133 | 0.6398 |
| Note:SNP:single nucleotide polymorphism.EA:effect allele. OA:other allele. | | | | | | | | | | | |
