# Supplementary material for: Genetically predicted obstructive sleep apnea is causally associated with an increased risk for periodontitis
Source: BMC Oral Health. 2023 Oct 6;23:723. doi: 10.1186/s12903-023-03338-8 (PMC10559524; doi:10.1186/s12903-023-03338-8)
Supplement: Supplementary file 6 — Supplementary Material 6 [file 12903_2023_3338_MOESM6_ESM.docx]

**STROBE-MR checklist of recommended items to address in reports of Mendelian randomization studies**^1^ ^2^

| **Item No.** | **Section** | **Checklist item** | **Page No.** | **Relevant text from manuscript** |
| --- | --- | --- | --- | --- |
| 1 | **TITLE and ABSTRACT** | Indicate Mendelian randomization (MR) as the study’s design in the title and/or the abstract if that is a main purpose of the study | 2 | Title of this article was ”Genetically predicted obstructive sleep apnea is causally associated with an increased risk for periodontitis”, and Mendelian randomization was described in its abstract as MR. |
|  | **INTRODUCTION** |  |  |  |
| 2 | **Background** | Explain the scientific background and rationale for the reported study. What is the exposure? Is a potential causal relationship between exposure and outcome plausible? Justify why MR is a helpful method to address the study question | 3-5 | Although several research have shown significant associations between periodontitis and OSA, it is nearly challenging to conduct randomized controlled trials to examine the association between OSA and periodontal outcomes, and there is still a lack of causal evidence for OSA and periodontitis. Therefore, MR is used to investigate whether OSA is associated to periodontitis in order to ultimately prevent or postpone the disease's progression. |
| 3 | **Objectives** | State specific objectives clearly, including pre-specified causal hypotheses (if any). State that MR is a method that, under specific assumptions, intends to estimate causal effects | 5 | The aim of the study was to explore the causal relationship between OSA and periodontitis using two-way MR. |
|  | **METHODS** |  |  |  |
| 4 | **Study design and data sources** | Present key elements of the study design early in the article. Consider including a table listing sources of data for all phases of the study. For each data source contributing to the analysis, describe the following: |  | In this study, genome-wide association studies (GWAS) data from the Gene-Lifestyle Interaction in the Dental Endpoints Consortium and FinnGen consortium were used. |
|  | a) | Setting: Describe the study design and the underlying population, if possible. Describe the setting, locations, and relevant dates, including periods of recruitment, exposure, follow-up, and data collection, when available. | 5-6 | Data from this GWAS analysis, were taken from the FinnGen consortium (R6). The PD summary statistics were obtained from the most recent meta-analysis of the GWAS of the Gene-Lifestyle Interaction in the Dental Endpoints Consortium. GWAS analyses were conducted on participants with European ancestry. |
|  | b) | Participants: Give the eligibility criteria, and the sources and methods of selection of participants. Report the sample size, and whether any power or sample size calculations were carried out prior to the main analysis | 5-6 | Data from this GWAS analysis, including 201,194 controls and 16,761 OSA cases. The PD summary statistics contains 17,353 clinically diagnosed cases and 28,210 controls. GWAS analyses were conducted on participants with European ancestry. In all of these summary genetic association estimates, the source articles contain more information on the population characteristics and particular trait definitions. |
|  | c) | Describe measurement, quality control and selection of genetic variants | 6-7 | SNPs were classified as IVs in the current investigation. For the first hypothesis, candidate IVs were created using summary statistics of OSA-associated SNPs with genome-wide significance (*p* < 5e −6). For each exposure, we used the clumping technique with R^2^ < 0.001 and a window size of 10,000 kb to filter SNPs that were in significant linkage disequilibrium to guarantee independence among instrumental factors. Then, SNPs with minor allele frequencies of <0.01 were removed. The F statistic was used as a measure of the strength of the IV of SNPs, calculated separately and cumulatively for each SNP via the formula F = R^2^× (N − 2)/(1 − R^2^). The F-statistics for each of these IVs needed to be >10, indicating that they fulfill the strong correlation requirement of MR. Considering the second hypothesis of MR, we used the PhenoScanner database（http://www.Phenoscanner.cam.ac.uk/）to identify SNPs with possible associations with confounders and remove them. After several rigorous screenings, the remaining SNPs were considered eligible IVs. |
|  | d) | For each exposure, outcome, and other relevant variables, describe methods of assessment and diagnostic criteria for diseases | 5-6 | The diagnosis of OSA was made in accordance with the International Classification of Diseases (ICD-10: G47.3, ICD-9: 3472A), which is based on patient reports of symptoms, clinical examination, and sleep registry using the apnea–hypopnea index or respiratory event index of 5/hour. Age, sex, genotyping chip, genetic relationship, and the first 10 main components of the analyses were all taken into account. Cases of periodontitis are defined by either Centers for Disease Control and Prevention/American Academy of Periodontology definitions(CDC/AAP)(24) or the Community Periodontal Index (CPI). |
|  | e) | Provide details of ethics committee approval and participant informed consent, if relevant | 6 | All the GWAS data involved in this study complied with the Declaration of Helsinki published in 1975 (amended in 2013), and each study received ethical approval from the local institutional ethics committees and informed consent of all patients. |
| 5 | **Assumptions** | Explicitly state the three core IV assumptions for the main analysis (relevance, independence and exclusion restriction) as well assumptions for any additional or sensitivity analysis | 5 | To obtain reliable results, IVs must satisfy three key assumptions in the MR analysis process: (1) Genetic variation should be significantly associated with exposure; (2) IVs should not be associated with any confounding factors affecting the exposure and outcome; and (3) IVs should affect outcome only indirectly through exposure factors and not through other pathways (no horizontal pleiotropy). |
| 6 | **Statistical methods: main analysis** | Describe statistical methods and statistics used |  |  |
|  | a) | Describe how quantitative variables were handled in the analyses (i.e., scale, units, model) | N/A | The exposure and outcome of this study were qualitative variables, and the treatment of quantitative variables was not involved. |
|  | b) | Describe how genetic variants were handled in the analyses and, if applicable, how their weights were selected | 6-7 | Candidate IVs were created using summary statistics of OSA-associated SNPs with genome-wide significance (*p* < 5e −6). For each exposure, we used the clumping technique with R^2^ < 0.001 and a window size of 10,000 kb to filter SNPs that were in significant linkage disequilibrium to guarantee independence among instrumental factors. Then, SNPs with minor allele frequencies of <0.01 were removed. The F statistic was used as a measure of the strength of the IV of SNPs, calculated separately and cumulatively for each SNP via the formula F = R^2^ × (N − 2)/(1 − R^2^). The F-statistics for each of these IVs needed to be >10, indicating that they fulfill the strong correlation requirement of MR. We used the PhenoScanner database（http://www.Phenoscanner.cam.ac.uk/）to identify SNPs with possible associations with confounders and remove them. After several rigorous screenings, the remaining SNPs were considered eligible IVs. |
|  | c) | Describe the MR estimator (e.g. two-stage least squares, Wald ratio) and related statistics. Detail the included covariates and, in case of two-sample MR, whether the same covariate set was used for adjustment in the two samples | 7-8 | To investigate the potential causal inferences between OSA and periodontitis, inverse variance weighting (IVW), MR-Egger regression, and weighted median (WM) were the three MR techniques used. The IVW method is the most commonly used method for MR analysis and is comparable to a weighted linear regression of the correlation between IV and outcome. The IVW method uses meta-analysis to combine the Wald estimates of each SNP for obtaining two-sample MR estimates of the association between OSA and periodontitis to evaluate causality. For estimating causality, the MR-Egger regression and WM method are also comparable to the IVW method. The slope of the MR-Egger represents the potential causal effect, on top of its intercept term, which facilitates the assessment of horizontal multiplicity. When there is evidence of pleiotropy, MR-Egger regressions are preferred. WM allows consistency analysis of multiple genetic instruments by calculating a single WM estimate; the results can provide reliable causal estimates even if up to 50% of SNPs are invalid genetic instruments. |
|  | d) | Explain how missing data were addressed | N/A | There were no missing data in this study. |
|  | e) | If applicable, indicate how multiple testing was addressed | N/A | Multiple testing was not involved in this study. |
| 7 | **Assessment of assumptions** | Describe any methods or prior knowledge used to assess the assumptions or justify their validity | 6-7 | The F-statistics for each of these IVs needed to be >10, indicating that they fulfill the strong correlation requirement of MR. |
| 8 | **Sensitivity analyses and additional analyses** | Describe any sensitivity analyses or additional analyses performed (e.g. comparison of effect estimates from different approaches, independent replication, bias analytic techniques, validation of instruments, simulations) | 8 | MR-Egger regression was performed to assess the likelihood of horizontal pleiotropy. The mean pleiotropic effect of IV was characterized by the intercept term of the MR-Egger regression. To correct horizontal pleiotropy by removing potential outliers, the MR-PRESSO was used as part of the pleiotropy analysis (Verbanck, Chen, Neale, & Do, 2018). The Cochran's Q test was used to verify the value of heterogeneity between the causal estimates of each SNP for the IVW and MR-Egger methods. There was no heterogeneity between IVs if the Q statistic was *p* > 0.1. Furthermore, a “leave-one-out” sensitivity analysis was performed to determine whether the potential effect of an SNP on the causal estimates was caused by a particular SNP and to verify the robustness and consistency of the findings. |
| 9 | **Software and pre-registration** |  |  |  |
|  | a) | Name statistical software and package(s), including version and settings used | 8 | “Two SampleMR” (Hemani et al., 2018) packages were used for all studies. A causal relationship was presumed to exist if an observed *P* < 0.05 supported it statistically. |
|  | b) | State whether the study protocol and details were pre-registered (as well as when and where) | N/A | The study protocol and details were not pre-registered. |
|  | **RESULTS** |  |  |  |
| 10 | **Descriptive data** |  |  |  |
|  | a) | Report the numbers of individuals at each stage of included studies and reasons for exclusion. Consider use of a flow diagram | 6 | The number of individuals is provided in the methodological section of this study. The source article contains specific information about the study population. |
|  | b) | Report summary statistics for phenotypic exposure(s), outcome(s), and other relevant variables (e.g. means, SDs, proportions) | 6 | The source articles contain more information on the population characteristics and particular trait definitions. |
|  | c) | If the data sources include meta-analyses of previous studies, provide the assessments of heterogeneity across these studies | 6 | The source articles contain more information on the assessments of heterogeneity. |
|  | d) | For two-sample MR:  i.  Provide justification of the similarity of the genetic variant-exposure associations between the exposure and outcome samples  ii.  Provide information on the number of individuals who overlap between the exposure and outcome studies | 6 | In order to reduce the ethnic heterogeneity caused by population stratification, the two sample populations in this study were from Europe. |
| 11 | **Main results** |  |  |  |
|  | a) | Report the associations between genetic variant and exposure, and between genetic variant and outcome, preferably on an interpretable scale | 9 | The F-statistics for these IVs were all >10, demonstrating that genetic variants are strongly correlated with exposure and outcome. |
|  | b) | Report MR estimates of the relationship between exposure and outcome, and the measures of uncertainty from the MR analysis, on an interpretable scale, such as odds ratio or relative risk per SD difference | 9 | MR Results are shown as odds ratio and 95% confidence intervals. The IVW method was used to assess a positive association between genetically predicted OSA and periodontitis, revealing that the risk of periodontitis was 1.117 times higher in individuals with OSA than in those without OSA (OR IVW = 1.117, 95% CI = 0.001–1.246, *p* = 0.048). Furthermore, MR assessments using MR-Egger estimates (OR MR-Egger = 1.305, 95% CIs = 0.859–1.983, *p* = 0.225) and WMs (OR WM = 1.105, 95% CIs = 0.95–1.281, *p* = 0.186) revealed generally consistent directions of effect, although they were typically rarely statistically significant, owing to the lower power of these two approaches. |
|  | c) | If relevant, consider translating estimates of relative risk into absolute risk for a meaningful time period | 9 | MR Results are shown as odds ratio and 95% confidence intervals. |
|  | d) | Consider plots to visualize results (e.g. forest plot, scatterplot of associations between genetic variants and outcome versus between genetic variants and exposure) |  | The Scatter plot and the funnel plot are shown in Figure 2 and Figure 3. |
| 12 | **Assessment of assumptions** |  |  |  |
|  | a) | Report the assessment of the validity of the assumptions | 9 | The F-statistics for these IVs (27.25125–85.66920) were all >10, demonstrating that there was no weak instrumental bias affecting the estimation of causal effects. |
|  | b) | Report any additional statistics (e.g., assessments of heterogeneity across genetic variants, such as *I^2^*, Q statistic or E-value) | 10 | The Cochran’s Q statistic found no proof of heterogeneity in causal inferences (MR-Egger *p* = 0.938 and IVW *p* = 0.943); |
| 13 | **Sensitivity analyses and additional analyses** |  |  |  |
|  | a) | Report any sensitivity analyses to assess the robustness of the main results to violations of the assumptions | 10 | The intercept term for the MR-Egger regression was calculated, which showed no horizontal pleiotropy among the seven selected SNPs (intercept = −0.013; standard error 0.018, *p* = 0.456). . |
|  | b) | Report results from other sensitivity analyses or additional analyses | 10 | The results of the leave-one-out analysis recommend that the association between OSA and periodontitis risk is not driven by individual SNPs, demonstrating that the MR results are robust and reliable. |
|  | c) | Report any assessment of direction of causal relationship (e.g., bidirectional MR) | 11-12 | Using the same method as forward MR analyses, According to the main findings of IVW, no statistical evidence linking an increased incidence of OSA to an increased risk of periodontitis was noted (OR = 1.00, 95% CI: 0.95–1.06, *p* = 0.87). Furthermore, consistent findings were obtained using the MR-Egger (OR = 1.00, 95% CI: 0.94–1.07, *p* = 0.98) and WM (OR = 0.99, 95% CI: 0.93–1.06, *p* = 0.78). A Cochran’s Q statistic evaluation of the SNPs revealed no evidence of heterogeneity between them (MR-Egger *p* = 0.605 IVW *p* = 0.723) (Table 2). The results of the MR-Egger intercept test ruled out any directional pleiotropy (intercept = 0.002; standard error = 0.009, *p* = 0.850). The leave-one-out sensitivity analysis showed that no particular SNP was significantly responsible for the association between OSA and periodontitis. |
|  | d) | When relevant, report and compare with estimates from non-MR analyses | 12 | Our MR research is in line with prior findings and supports the hypothesis that the increased incidence of periodontitis owing to OSA is driven by a causative impact. |
|  | e) | Consider additional plots to visualize results (e.g., leave-one-out analyses) |  | Figure 3 |
|  | **DISCUSSION** |  |  |  |
| 14 | **Key results** | Summarize key results with reference to study objectives | 12 | Our findings provide credibility to the idea that OSA causatively affects periodontitis. A reverse MR study was also conducted, but no evidence suggested periodontitis as a cause of OSA. |
| 15 | **Limitations** | Discuss limitations of the study, taking into account the validity of the IV assumptions, other sources of potential bias, and imprecision. Discuss both direction and magnitude of any potential bias and any efforts to address them | 14-15 | Nevertheless, the present study has several limitations. First, In MR, the most widely adopted approach relies on the inference of SNPS identified by genome-wide association studies (GWAS). Given the variations in quality control when conducting individual GWAS, it was difficult to offset the potential confounding bias that would have affected the results. Second, our findings only reflect the life-long impact of OSA on periodontitis. However, the short-term effect of OSA on the risk of periodontitis remains unknown. Third, given our population restriction to European ancestry, the conclusions cannot be generalized to other populations. Additionally, since the research used pooled data, further stratified data analyses based on individual-level data are needed to further examine the effect of the causal link between OSA and periodontitis. Finally, due to the inconsistent definition of periodontitis used in various studies and the numerous challenges faced during disease analysis, GWAS for periodontitis tends to fail to discover consistent SNPs. Future high-quality GWAS are still required to further study the potential etiological role of OSA in periodontitis. |
| 16 | **Interpretation** |  |  |  |
|  | a) | Meaning: Give a cautious overall interpretation of results in the context of their limitations and in comparison with other studies | 12 | Observational research has recently shown that OSA may play a critical role in the development and progression of periodontitis. A case-control study using conditional logistic regression analysis on 7673 patients with OSA and 21963 controls demonstrated a relationship between OSA and previously identified periodontitis (Keller, Wu, Chen, & Lin, 2013). Similar conclusions have been obtained by stratification studies conducted for different age groups. Based on the findings of a cross-sectional study conducted in Korea, patients with OSA aged ≥55 years have more than double the risk of developing chronic periodontitis than healthy controls (Seo et al., 2013). Another large population-based study including Hispanic/Latino adults further showed that breathing disorders during sleep and severe periodontitis in young adults are positively associated (Seo et al., 2013). Furthermore, results from observational studies in several countries, including Jordan, India, and the United States, have shown that OSA and a higher risk of periodontitis are associated, suggesting that OSA is a potential predictor of periodontitis (Al Habashneh, Khassawneh, Khader, Abu-Jamous, & Kowolik, 2016; Latorre et al., 2018; Mukherjee & Galgali, 2021). Similarly, a meta-analysis further confirmed this (Al-Jewair, Al-Jasser, & Almas, 2015; Khodadadi et al., 2022; Zhang et al., 2022; Zhu et al., 2022). Our MR research is in line with prior findings and supports the hypothesis that the increased incidence of periodontitis owing to OSA is driven by a causative impact. |
|  | b) | Mechanism: Discuss underlying biological mechanisms that could drive a potential causal relationship between the investigated exposure and the outcome, and whether the gene-environment equivalence assumption is reasonable. Use causal language carefully, clarifying that IV estimates may provide causal effects only under certain assumptions | 13-14 | To clarify the association between OSA and periodontitis, several explanations have been proposed in prior research. First, mouth breathing is a potential cause of periodontal diseases. Oral breathing is a habitual behavior of patients with OSA. Prolonged mouth opening may alter the oral environment and hinder the mouth's natural cleansing processes, facilitating greater colonization of the periodontal microbiota and subsequent development of periodontitis (Koutsourelakis, Vagiakis, Roussos, & Zakynthinos, 2006; Oeverland, Akre, & Skatvedt, 2002). Second, OSA is often associated with intermittent hypoxia (IH). OSA can encourage transcription factors such as nuclear factor due to intermittent hypoxia, causing increased production of pro-inflammatory cytokines that can aggravate the inflammatory condition of preexisting disease and even start inflammatory illness in the host, thus causing an augmented risk of periodontitis and its severity (Chen et al., 2021). In vivo studies have shown that intermittent hypoxia in OSA might affect immune transcription factors such as host HIF-1, which can considerably increase bone mineral density and modify bone microstructure, which is a possible risk factor for poor autostasis in developing alveolar bone (Oishi et al., 2016; Schaffer & Taylor, 2015). Furthermore, oxidative stress is an additional factor that contributes to OSA-related periodontal tissue damage. The mechanism of this includes encouraging the generation of reactive oxygen species and oxygen free radicals, leading to the formation of local and systemic inflammatory responses (Lavie, 2015). Subgingival plaque and saliva-serum cytokine levels were measured in patients with OSA in a study; OSA was found to be associated with worsening periodontal disease and greater amounts of IL-6 and apelin in the saliva, as well as changed the bacteria that were examined in plaque (Nizam et al., 2015; Nizam et al., 2016; Nizam et al., 2014). Furthermore, by changing the microbial community around the periodontium, OSA can initiate the development of periodontitis. A study using 16S rRNA sequencing revealed that the species richness and trans-habitat diversity of the salivary microbial community were altered in the OSA group, and coupled with Prevotella (a particular periodontal pathogen), it showed an increase in the tendency of OSA in patients (Chen et al., 2021). These findings offer fresh insights into the pathophysiology of periodontitis. |
|  | c) | Clinical relevance: Discuss whether the results have clinical or public policy relevance, and to what extent they inform effect sizes of possible interventions | 15 | Moreover, we suggest that early detection and management of OSA can be a new strategy to improve periodontitis in the future. |
| 17 | **Generalizability** | Discuss the generalizability of the study results (a) to other populations, (b) across other exposure periods/timings, and (c) across other levels of exposure | 14 | Given our population restriction to European ancestry, the conclusions cannot be generalized to other populations. Additionally, since the research used pooled data, further stratified data analyses based on individual-level data are needed to further examine the effect of the causal link between OSA and periodontitis. |
|  | **OTHER INFORMATION** |  |  |  |
| 18 | **Funding** | Describe sources of funding and the role of funders in the present study and, if applicable, sources of funding for the databases and original study or studies on which the present study is based | 16 | This study was supported by the Local Science and Technology Development Fund Project guided by the Central Government of Shanxi Provincial Department of Science and Technlogy, Grant/Award Number; YDZX20201400001131, and the Science and Technology Innovation Project of Higher Education Institutions of Shanxi Province, Grant; 2022L167. |
| 19 | **Data and data sharing** | Provide the data used to perform all analyses or report where and how the data can be accessed, and reference these sources in the article. Provide the statistical code needed to reproduce the results in the article, or report whether the code is publicly accessible and if so, where | 15-16 | The data used for analysis were obtained from published studies and public databases. All data generated during this study are included in this article and supple-mentary materials. |
| 20 | **Conflicts of Interest** | All authors should declare all potential conflicts of interest | 15 | The authors declare no conflicts of interest. |

This checklist is copyrighted by the Equator Network under the Creative Commons Attribution 3.0 Unported (CC BY 3.0) license.

1. Skrivankova VW, Richmond RC, Woolf BAR, Yarmolinsky J, Davies NM, Swanson SA, et al. Strengthening the Reporting of Observational Studies in Epidemiology using Mendelian Randomization (STROBE-MR) Statement. JAMA. 2021;under review.

2. Skrivankova VW, Richmond RC, Woolf BAR, Davies NM, Swanson SA, VanderWeele TJ, et al. Strengthening the Reporting of Observational Studies in Epidemiology using Mendelian Randomisation (STROBE-MR): Explanation and Elaboration. BMJ. 2021;375:n2233.
