# Supplementary material for: Genetically predicted obstructive sleep apnea is causally associated with an increased risk for periodontitis
Source: BMC Oral Health. 2023 Oct 6;23:723. doi: 10.1186/s12903-023-03338-8 (PMC10559524; doi:10.1186/s12903-023-03338-8)
Supplement: Supplementary file 7 — Supplementary Material 7 [file 12903_2023_3338_MOESM7_ESM.docx]

**Supplementary Figure Legends**

**Supp.Fig.S1** Scatter plot depicts the causal estimates of the effect of periodontitis on OSA. Each point in the scatter plot represents an SNP. The effect of the same SNP on exposure is placed on the horizontal axis, and the effect on outcome is placed on the vertical axis. The vertical and horizontal lines show the 95% confidence interval (CI) for each SNP. At this point, the slope of the solid line in the plot is each Mendelian randomization (MR) estimate. OSA, obstructive sleep apnea; SNP, single nucleotide polymorphism.

**Supp.Fig.S2** The funnel plot assesses the possible heterogeneity in the estimates. Each point indicates the inverse standard error corresponding to the individual causal estimates. The mean causal effect of all combinations of IVs (βIV) is indicated on the X-axis by inverse variance weighting (solid line) and the Mendelian randomization (MR)-Egger method (dashed line). The Y-axis indicates the inverse standard error of the estimated causal effect for each single nucleotide polymorphism (IVs).

**Supp.Fig.S3** The leave-one-out plot present how the causal estimates (point with horizontal circle) for the effect of periodontitis on OSA were influenced by the removal of a single variant. The bars indicate the confidence interval of MR estimates. OSA, obstructive sleep apnea; SNP, single nucleotide polymorphism.
